# Predicting the Risk of Loneliness in Children and Adolescents: A Machine Learning Study

**DOI:** 10.3390/bs14100947

**Published:** 2024-10-15

**Authors:** Jie Zhang, Xinyi Feng, Wenhe Wang, Shudan Liu, Qin Zhang, Di Wu, Qin Liu

**Affiliations:** 1 Research Center for Medicine and Social Development, School of Public Health, Chongqing Medical University, No. 1 Yixueyuan Road, Yuzhong District, Chongqing 400016, China191095@cqmu.edu.cn (S.L.);; 2 College of Medical Informatics, Chongqing Medical University, No. 1 Yixueyuan Road, Yuzhong District, Chongqing 400016, China

**Keywords:** loneliness, machine learning, child and adolescent, prediction, mental health

## Abstract

Background: Loneliness is increasingly emerging as a significant public health problem in children and adolescents. Predicting loneliness and finding its risk factors in children and adolescents is lacking and necessary, and would greatly help determine intervention actions. Objective: This study aimed to find appropriate machine learning techniques to predict loneliness and its associated risk factors among schoolchildren. Methods: The data were collected from an ongoing prospective puberty cohort that was established in Chongqing, Southwest China. This study used 822 subjects (46.84% boys, age range: 11–16) followed in 2019. Five models, (a) random forest, (b) extreme gradient boosting (XGBoost), (c) logistic regression, (d) neural network, and (e) support vector machine were applied to predict loneliness. A total of 39 indicators were collected and 28 predictors were finally included for prediction after data pre-processing, including demographic, parental relationship, mental health, pubertal development, behaviors, and environmental factors. Model performance was determined by accuracy and AUC. Additionally, random forest and XGBoost were applied to identify the important factors. The XGBoost algorithm with SHAP was also used to interpret the results of our ML model. Results: All machine learning performed with favorable accuracy. Compared to random forest (AUC: 0.87 (95%CI: 0.80, 0.93)), logistic regression (AUC: 0.80 (95%CI: 0.70, 0.89)), neural network (AUC: 0.80 (95%CI: 0.71, 0.89)), and support vector machine (AUC: 0.79 (95%CI: 0.79, 0.89)), XGBoost algorithm had the highest AUC values 0.87 (95%CI: 0.80, 0.93) in the test set, although the difference was not significant between models. Peer communication, index of general affect, peer alienation, and internet addiction were the top four significant factors of loneliness in children and adolescents. Conclusions: The results of this study suggest that machine learning has considerable potential to predict loneliness in children. This may be valuable for the early identification and intervention of loneliness.

## 1. Introduction

Loneliness is a negative, subjective experience characterized by a discrepancy between an individual’s aspirations and their actual level of social relationships [1]. Subjective loneliness differs from objective social isolation; individuals with numerous social contacts can still experience feelings of loneliness. Loneliness is increasingly recognized as a significant public health concern [2]. While existing research and interventions primarily target the elderly, there is increasing attention on loneliness among children and adolescents. A scoping review indicates that in some countries with loneliness intervention policies, children and adolescents are included as target groups alongside older adults [3]. Moreover, loneliness exhibits a U-shaped trajectory across the lifespan, with a higher prevalence among adolescents [4]. A recent meta-analysis of loneliness in 113 countries found that the prevalence of loneliness among adolescents (12–17 years) ranged from 9.2% to 14.4% [5].

Like the profound loneliness experienced by older adults, loneliness in children and adolescents is linked to various negative health outcomes. Prior research has established a positive correlation between loneliness and mental health issues in children and adolescents, particularly concerning depression and anxiety [6]. Recently, David et al. conducted a genome-wide study that demonstrated a causal relationship between loneliness and depression [7]. Additional studies have highlighted connections between loneliness and maladaptive behaviors such as sleep [8], substance use [9], and even suicidal ideation [10] among children and adolescents. Importantly, findings parallel those observed in older populations, indicating that loneliness is also related to physical health issues, such as cardiovascular disease, which may be independent from the effect of depression [11]. Furthermore, childhood loneliness may incur direct economic costs, potentially influencing employment prospects in adulthood [12]. Given that the prevalence of loneliness among adolescents may still be increasing [13], and its associated severe health outcomes, appropriate approaches are needed to predict and intervene in cases of loneliness [14].

### 1.1. Risk Factors for Loneliness in Children and Adolescents

The association between risk factors and loneliness differs quite substantially, including demographic, psychological, and social factors. Furthermore, many of these risk factors are complex and interrelated. In adults, being married, in a partnership, or sharing a household with someone serves as a protective factor, particularly for men [15]. Recent studies have also underscored the importance of biological variables in understanding loneliness from an evolutionary perspective, especially concerning epigenetic research [16]. In children and adolescents, the relevant factors may differ. Peer relationships may play a significant role in loneliness [17,18] as the social relationship of children is redirected from parents to peers during adolescence [19]. Liu et al. found that the influence of peer relationships on loneliness may be mediated by interactions between environmental factors and genetic predispositions [20]. Since adolescents are exposed to more screen time compared to adults, research [21] has shown that internet addiction raises loneliness in children and adolescents. Additionally, well-being [22] and obesity [23] may also be related to loneliness in children and adolescents. However, those existing studies are mostly based on traditional statistical models programmed by a human to deliver a fixed solution, which may pose difficulty in addressing the complex relationships (e.g., nonlinear relationships) between a large number of risk factors and have limitations in determining which risk factor is most significant. In contrast, machine learning techniques for classification and prediction modeling have the advantage of being able to handle complex relationships (high-dimensional relationships) between variables that may not have been identified and can handle large quantities of data [24].

### 1.2. Machine Learning (ML) in Predicting Loneliness

Machine learning (ML) has been effectively utilized in mental health for both prediction and treatment, achieving excellent accuracy [25]. Recently, various ML methods, such as random forest (RF)—an ensemble algorithm based on decision trees—and support vector machines, have been employed to predict depression, anxiety [26], suicide [27], and their risk factors in children and adolescents. However, there is a notable lack of studies focusing specifically on loneliness [28]. In adults, MLs have been applied to prediction of loneliness using data from social media [29] and wearable technologies. For instance, Afsaneh Doryab et al. [30] used gradient boosting and logistic regression algorithms with smartphone and Fitbit data to predict loneliness in 160 college students, achieving an accuracy of 80.2%. Therefore, it is an urgent need to develop effective models for predicting loneliness in children and adolescents at high risk, as this area remains underexplored. Additionally, further investigation into ML models that utilize a broader range of data sources, rather than relying solely on smartphone data, is essential.

This study aimed to find appropriate machine learning techniques to predict the risk of loneliness among schoolchildren. We assessed the accuracy of five machine learning techniques in predicting loneliness and explored the ranking of the importance of risk factors (including peer relationships, internet addiction, well-being, obesity, and other variables) for loneliness among schoolchildren.

## 2. Material and Methods

### 2.1. Data Set and Measures

The data were collected from an ongoing prospective puberty cohort that was established in Chongqing, Southwest China in 2014. Specifically, questionnaires and physical examination records were obtained from students at four schools within the cohort. Details of the cohort methodology have been reported previously [31]. The present study uses the data of the 11th follow-up visit with 822 students (46.84% boys, age range: 11–16) from the longitudinal study conducted in November 2019. Participants were recruited with informed consent from both their parents and themselves. This study received approval from the Medical Ethics Review Committee of Chongqing Medical University.

Loneliness was measured using the Child Loneliness Scale (CLS) [32], a tool widely utilized for younger children. This scale has demonstrated good reliability and validity compared to other measures of loneliness [33]. Each response is rated on a 5-point scale (from 1  =  never to 5  =  always), resulting in a total score that ranges from 16 to 80, where a higher score indicates greater levels of loneliness. When the total score is 46 or higher, loneliness is considered. Cronbach’s alpha is a statistical measure used to assess the reliability or internal consistency of a set of items in a test or questionnaire. It indicates how closely related the items are as a group, with values ranging from 0 to 1. A higher value suggests greater reliability; typically, above 0.7 is considered acceptable for research purposes [34]. In the present study, the Cronbach’s alpha of this scale was 0.915 (95%CI: 0.905, 0.924).

The inventory of parent and peer attachment was used to measure individuals’ peer attachment [35]. This scale consists of 25 items divided into three subscales: peer trust (adolescents trust that peers understand and respect their needs and desires, e.g., “My friends understand me”), peer communication (adolescents’ perceptions that peers are sensitive and responsive to their emotional states and assessing the extent and quality of involvement and verbal communication with them, e.g., “When we discuss things‚ my friends care about my point of view”), and peer alienation (adolescents’ feelings of isolation, anger, and detachment experienced in attachment relationships with peers, e.g., “It seems as if my friends are irritated with me for no reason”). This study used calculated scores for peer trust (10 items), peer communication (8 items), and peer alienation (7 items). The Cronbach’s alpha of this scale was 0.891 (95%CI: 0.874, 0.903) in this study.

Subjective well-being was assessed using the well-being index developed by Campbell [36]. The scale includes two components: the index of general affect, which consists of eight emotional items, and the index of life satisfaction, comprising two items related to life satisfaction. In this study, the Cronbach’s alpha for this scale was 0.954 (95%CI: 0.947, 0.959).

Internet addiction was measured using the Internet Addiction Test developed by Young [37], based on the DSM-IV diagnostic criteria for pathological gambling. This scale contains 20 items, with the Chinese version scored from 0 to 5, where higher scores indicate more severe symptoms. The total score of the scale was utilized for analysis in this study. The Cronbach’s alpha for this scale was 0.922 (95%CI: 0.914, 0.930).

**Figure 1:**
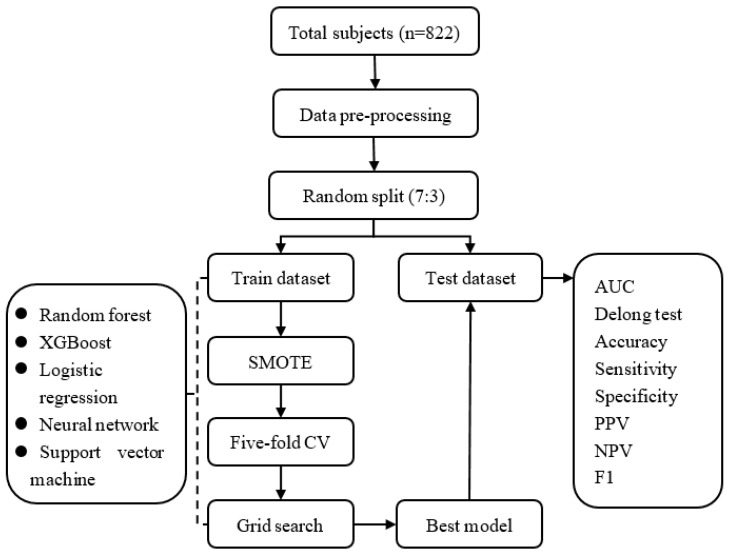
The study flowchart. SMOTE: Synthetic Minority Over-sampling Technique; AUC: area under the receiver operating characteristic curve; PPV: positive predictive value; NPV: negative predictive value; F1: F1 score.

Other variables included sociodemographic variables (gender, age, etc.), school-related variables (grade, class, etc.), family-related variables (parental marital status, whether parents work outside the home, family size, etc.) from questionnaires, and physical measurements (height, weight, waist circumference, pubertal developmental characteristics, etc.). Overall, a total of 39 variables were included. In this study, a total of 822 study subjects were included in the analysis with an average age of 13.52 years. The participants were from grades 7 to 10, including 385 (46.84%) males. The overall prevalence of loneliness was 109 (13.26%). Other basic variable information can be found in Table 1. Referring to recent clinical machine learning guidelines [38], we designed the model development and evaluation process for the entire study. The whole study flowchart is shown in Figure 1.

### 2.2. Statistical Analysis

#### 2.2.1. Data Pre-Processing

Variables with missing rates exceeding 20% were removed from the analysis (1 variable excluded). Additionally, variables with zero variance were eliminated (5 variables excluded). For independent variables with a correlation coefficient greater than 0.8, only one variable was retained (4 variables excluded). Ultimately, a total of 29 variables were deemed eligible for inclusion in the analysis. Excluding loneliness, these 28 predictors included grade, class, gender, parental relationship (relationship between parents, only child, father works in another city, number of persons living together, and parents’ marital status), pubertal development (sexual characteristics, self-reported height growth, self-reported level of development, and knowledge of puberty), mental health (peer communication, peer alienation, internet addiction, index of general affect, and index of life satisfaction), physical measurements (height and waist circumference), behaviors (weekday screen time, weekend screen time, and myopia), environmental factors (secondhand smoke, whether to use camphor pills, insecticide, air freshener, use of cosmetics, and whether to use hair straightening cream). The variables and their values used in the machine learning model are provided in the Supplementary Materials. Due to the requirement of complete datasets for most machine learning techniques, missing values were imputed using RF implemented with the R package missForest.

#### 2.2.2. Model Development

We split data into a training set (70% of the sample) and a test set (30% of the sample). Given that positive cases were rare at only 13.3%, we employed the Synthetic Minority Over-sampling Technique (SMOTE) to address this issue in the training set, using the R package DMwR [39]. Based on existing systematic reviews of machine learning in mental health [40], we selected the five most commonly and effectively used machine learning models. These models include random forest (RF), XGBoost, logistic regression, neural networks, and support vector machines. We utilized the caret R package to build these predictive models, with classifier information detailed in Table 2 [41]. Additionally, we implemented 5-fold cross-validation (5-fold CV) and hyperparameter tuning to identify the final model. Hyperparameters are parameters in machine learning models that need to be set manually, typically defined before the model training begins. These parameters cannot be automatically adjusted during the training process and must be determined based on experience, experimentation, or algorithm selection. The choice of hyperparameters has a significant impact on the model’s performance. For example, by appropriately lowering mtry (the number of features considered for each tree) in a random forest, one can reduce the model’s complexity, prevent overfitting, and improve the model’s generalization ability [42]. The parameter tuning part was guided by standard practice, and models except logistic regression were used to grid search for model hyperparameter tuning using the R package caret; see Table 3.

All analyses were conducted using R version 4.2.2.1 (The R Foundation for Statistical Computing, Vienna, Austria), and the final model was fitted to the test set. Variable importance was assessed for both random forest (RF) and XGBoost. The importance of RF was evaluated with Mean Decrease Gini (MDG), which calculates the effect of each variable on the heterogeneity of observations at each node of the classification tree [48], while the evaluation metric of XGBoost is Gain. Gain indicates the relative contribution of each feature to the model by measuring their contribution in each tree. The higher the values of either MDG or Gain, the more important the feature is. To interpret the results of our ML model, we also combined the XGBoost algorithm with SHAP (Shapley Additive Explanations) by R package shapviz. SHAP values are a game theory-based tool for elucidating machine learning model outputs [49]. They quantify each feature’s contribution to predictions by assessing their impact across various combinations. SHAP values can also show the positive or negative contribution of each predictor variable to the target variable.

#### 2.2.3. Model Evaluation

In this study, the area under the receiver operating characteristic curve (AUC), the best performance metric of machine learning algorithms, was implemented to evaluate the models. A higher AUC value indicates a more effective classifier, whereas a value below 0.5 indicates weak performance or inaccurate predictions [50]. Confidence intervals at 95% were generated for each AUC by bootstrapping. The DeLong test was also employed to compare the differences between the ROC curves of different models. We also reported accuracy, sensitivity, specificity, positive and negative predictive value, and F1 score. Accuracy is defined as (Accuracy = (TP + TN)/(TP + TN + FP + FN)), where TP refers to true positives, TN to true negatives, FP to false positives, and FN to false negatives. This metric provides a straightforward assessment of overall performance but may not be reliable in cases of class imbalance. Sensitivity (also known as recall) is expressed as (Sensitivity = (TP)/(TP + FN)) and assesses the proportion of actual positive samples that are correctly identified, emphasizing the model’s ability to capture positive cases effectively. Specificity assesses the model’s ability to correctly identify negative instances, calculated as (Specificity = (TN)/(TN + FP)). The positive predictive value (PPV), also referred to as precision, reflects the proportion of true positive results in all positive predictions, expressed as (PPV = (TP)/(TP + FP)). Conversely, the negative predictive value (NPV) measures the proportion of true negative results in all negative predictions, given by (NPV = (TN)/(TN + FN)). The F1 score, calculated as (F1 Score = 2∗(TP/(TP + FP))∗(TP/(TP + FN))/(TP/(TP + FP) + TP/(TP + FN))), combines precision and recall into a harmonic mean, offering a balanced view of a model’s performance, particularly in imbalanced datasets.

## 3. Results

### 3.1. Prediction of Loneliness

**Figure 2:**
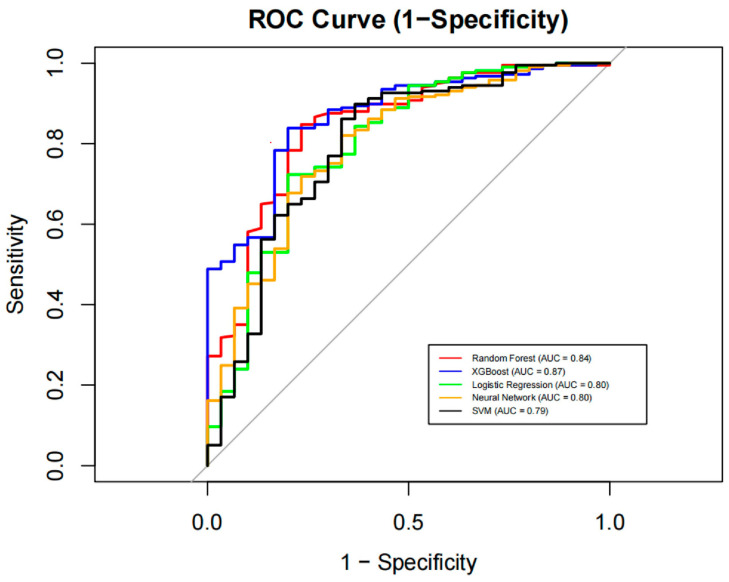
ROC curves for six machine learning models in predicting loneliness. Note: Five-fold cross-validation was used to construct and evaluate the predictive models. Different colors indicate different machine learning classifiers used in this study.

**Figure 3:**
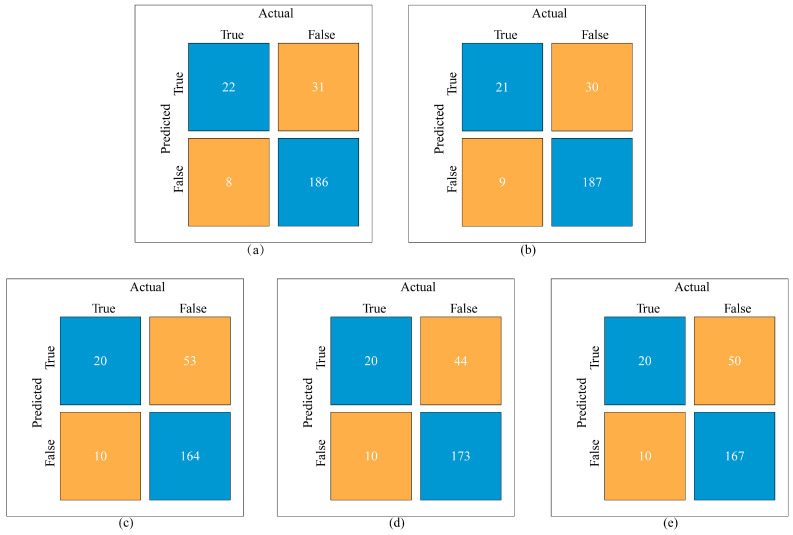
The confusion matrixes: random forest (**a**), XGBoost (**b**), logistic regression (**c**), neural network (**d**), support vector machine (**e**). The blue represents the number of true positives (TP, where both predicted and actual are true) and true negatives (TN, where both predicted and actual are false), while the yellow represents the number of false positives (FP, where the prediction is true and the actual is false) and false negatives (FN, where the prediction is false and the actual is true).

After hyperparameter tuning, the XGBoost algorithm had the highest AUC values 0.87 (95%CI: 0.80, 0.93) in the test set compared to RF (AUC: 0.87 (95%CI: 0.80, 0.93)), logistic regression (AUC: 0.80 (95%CI: 0.70, 0.89)), neural network (AUC: 0.80 (95%CI: 0.71, 0.89)), and support vector machine (AUC: 0.79 (95%CI: 0.79, 0.89)), although the difference was not significant in the DeLong test. RF predicted loneliness with the highest accuracy (0.84 (95%CI: 0.79, 0.89)), sensitivity (0.73), specificity (0.86), PPV (0.42), and NPV (0.96). Table 4 shows the other model’s performance. The classification performance of the six ML models based on 28 features is also shown in an ROC curve: XGBoost outperformed other models in terms of performance (Figure 2). Results from the confusion matrixes (Figure 3) indicate that the differences in prediction performance among the models primarily lie in their ability to predict negative outcomes.

### 3.2. Important Features

**Figure 4:**
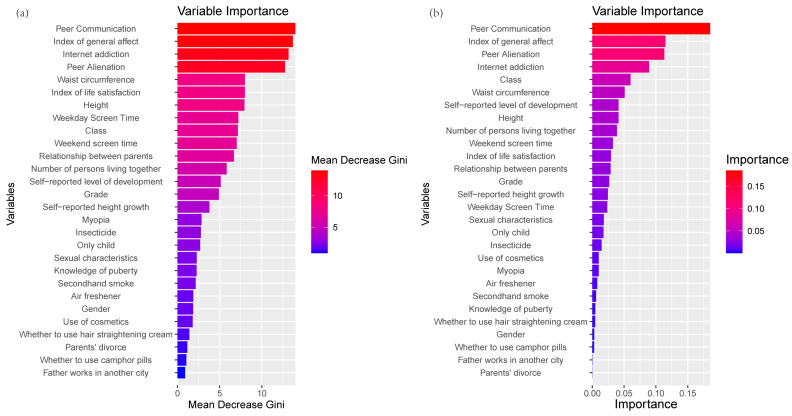
Variable importance in random forest (**a**) and XGBoost (**b**). The importance of RF was evaluated with Mean Decrease Gini (MDG), which calculates the effect of each variable on the heterogeneity of observations at each node of the classification tree. The evaluation metric of XGBoost is Gain, which indicates the relative contribution of each feature to the model by measuring their contribution in each tree.

**Figure 5:**
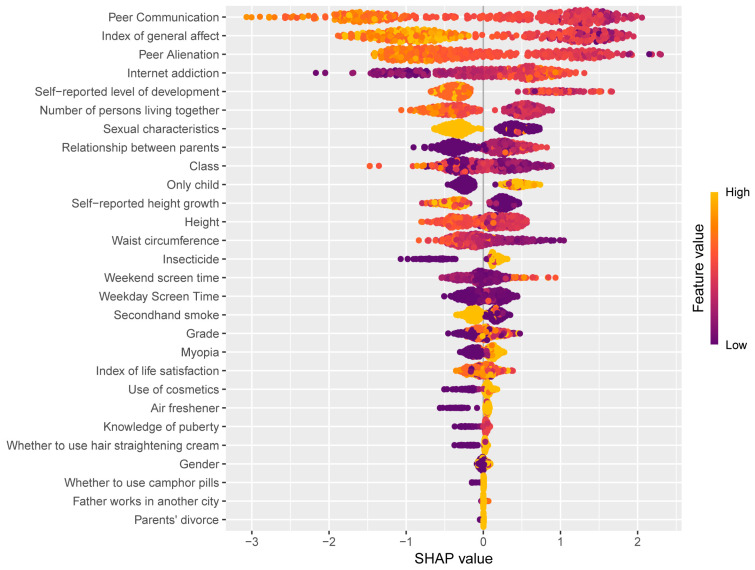
SHAP feature importance summary plot for XGBoost model. The SHAP plot provides a detailed visualization of how key features influence the model’s output for each individual. Each dot represents a distinct feature attribution value, with its vertical position indicating the feature’s relative importance and its horizontal position reflecting the SHAP value, which shows the variable’s contribution to the outcome. Dots are colored based on the original feature values—yellow for high values and purple for low—and accumulate vertically to illustrate density.

RF and XGBoost were applied to identify the important factors. Both models revealed the same top four important items: peer communication, index of general affect, peer alienation, and internet addiction (Figure 4). In the SHAP plot (Figure 5), peer communication, index of general affect, and peer alienation were negatively correlated with loneliness, while internet addiction was positively correlated with loneliness.

## 4. Discussion

An increasing number of studies emphasize the importance of loneliness in the health of children and adolescents. While loneliness is not yet classified as a disease, it may have unique neurobiological mechanisms associated with structural and functional changes in specific brain regions and networks [51]. Even though there are existing studies about loneliness prevalence and mechanism, studies on the prediction of loneliness are still lacking.

### 4.1. Model Performance

In our study, the prevalence of loneliness in children and adolescents was 13.3%, which was higher than the overall prevalence of loneliness (11.7%) among the 248,017 students from 70 countries [52]. Moreover, utilizing a wide range of data from questionnaires and physical examinations, this study effectively predicted loneliness in children and adolescents. Although the difference was not significant, XGBoost achieved the highest AUC value 0.87 (95%CI: 0.80, 0.93) in the test set. Notably, RF also demonstrated strong predictive performance in other metrics, including accuracy, precision, sensitivity, and positive and negative predictive values. Consistent with previous studies, XGBoost and RF have recently grown in significance in the diagnosis and prognosis of a variety of psychiatric and neurological conditions, including depression [53], Alzheimer’s disease [54], and anxiety [55]. Both methods are tree-based learning techniques that may help address the issue of class imbalance in loneliness, where the number of lonely individuals is smaller compared to those who are not, affecting the effectiveness of machine learning [56]. XGBoost can better focus on hard-to-classify minority samples by adjusting the learning rate and the structure of the trees, while random forest enhances the recognition of minority classes through the construction of multiple decision trees and a voting mechanism. Existing research also indicates that the SMOTE method combined with XGBoost performs well in handling imbalanced samples [57].

However, comparing the performance between machine learning and traditional models is challenging due to the lack of machine learning studies focusing on the prediction of loneliness in children and adolescents. First, machine learning has been studied more in the prediction of suicidal behavior in children and adolescents [58,59]. Second, there is also ongoing debate regarding whether machine learning outperforms traditional regression models in the existing literature. Van Mens et al. [60] found that machine learning did not result in superior performance over regular logistical regression, while Walsh et al. [61] found RF model significantly outperformed logistic regression. An earlier study [62] also indicated that traditional models are easier to explain with fewer variables, while the addition of more variables enhances the predictive power of machine learning models.

Notably, our model demonstrates high sensitivity and specificity, which are among the highest levels reported in existing machine learning studies for mental health [40,63]. This indicates its potential in identifying and excluding loneliness. Ideally, a screening model should have both high positive predictive value (PPV) and negative predictive value (NPV), but achieving this balance is often challenging and typically requires trade-offs. Our study demonstrates strong performance in NPV, though the PPV is less robust; however, it is still higher than in certain other machine learning studies [27], suggesting practical significance for screening by identifying individuals who may need further diagnostic tests [64]. Additionally, compared to mobile data-based machine learning research, our PPV is relatively lower, likely due to differences in the prevalence of loneliness. For PPV, the prevalence of positive cases is crucial [65]; the lower the prevalence, the more difficult it is to correctly predict positive cases. In the mobile data-based study, the prevalence of loneliness among university students was relatively balanced, with a higher prevalence of loneliness, whereas our sample was smaller, complicating the prediction of positive cases for loneliness.

### 4.2. Variable Importance

In the variable selection, among 28 features, peer communication was the most important feature across all models. This result is consistent with previous studies [66]. Peer relationships are particularly important in adolescent psychological development. A recent meta-analysis [67] reported that there were concurrent and longitudinal associations between peer relationships with loneliness in children and adolescents. This association was even found to be more stable when compared to the relationship between peer relationships and depression [67]. A study based on adolescence in Taiwan also found that good peer relationships in adolescence can reduce the incidence of loneliness in adulthood [68]. While studies have already proposed improving loneliness from the perspective of peer relationships within the elderly population [69], future research should consider implementing public health measures through peer relationships to reduce loneliness among children and adolescents.

Internet addiction is also important in constructing predictive models. Interestingly, internet reduces loneliness in older people [70], which may be reversed in children and adolescents. The reason might be that younger people are more likely to have problematic internet use [71] and even internet addiction. According to a meta-analysis [21], internet addiction and loneliness were found to be positively related. Previous studies [72] have found a further bidirectional relationship between internet addiction and loneliness in children and adolescents, with the lonelier being more prone to internet addiction. This suggests the importance of proper use of the internet in children and adolescents and controlling internet addiction.

Subjective well-being (index of general affect) also plays an important role in constructing predictive models. However, the relationship between subjective well-being and loneliness has primarily been examined in the context of older adults. VanderWeele et al. [73] reported that subjective well-being can predict loneliness and vice versa in older people even after controlling for depressive symptoms, social support, and psychiatric conditions and medications as time-varying confounders. Our study found that subjective well-being was equally important in the prediction of loneliness in children and adolescents. Future studies could explore this relationship further in children and adolescents.

### 4.3. Strengths and Limitations

To our knowledge, this is the first study to predict loneliness using machine learning methods in children and adolescents. We compared machine learning methods with traditional logistic regression and then developed potential machine learning models to predict loneliness in children and adolescents. Our data, derived from questionnaires and physical examinations, allowed for us to identify a wide range of risk factors associated with loneliness in this population. Additionally, we ranked the importance of these factors rather than merely exploring them.

There are also some limitations to this study: First, the findings may not be generalizable beyond Chongqing, China, as all participants were drawn from this specific region. Therefore, further external validation is needed to establish the applicability of our results in different contexts. Additionally, our research is based on a cross-sectional design, which restricts our ability to make causal inferences; longitudinal studies are essential for validating our findings over time. Another limitation is that the use of different thresholds in our analysis could yield varying results. However, our primary objective was not to determine the optimal threshold but to understand the loneliness experiences of children and adolescents and to assess the feasibility of applying machine learning approaches to predict loneliness. Moreover, the absence of biological indicators, which are important in understanding loneliness, may also limit the predictive power of our machine learning models. This omission could result in an underestimation of their effectiveness in identifying loneliness in this demographic. Finally, cultural aspects may potentially influence the results and provide valuable insights into the explanations of our findings. The previous literature suggests that individualism may increase loneliness in adults more than collectivism culture [74,75,76]. The role that culture plays in children’s loneliness also needs to be explored further. Although our study focuses on a single region with similar culture, it is necessary to study the association between culture and loneliness in children and adolescents in different regions.

### 4.4. Future Directions

Future studies should validate our findings in external populations with larger sample sizes. In addition, validation could be carried out on certain special populations in order to be more suitable for clinical applications. Moreover, the performance of machine learning can be explored at different threshold scales. Finally, future research from other places and ethnicities may be also required to explore the ability of ML to predict loneliness in different cultures.

## 5. Conclusions

We enhance the understanding of machine learning methods for predicting loneliness in children and adolescents using population-based data. Our study demonstrates that the established machine learning model effectively identifies children and adolescents at high risk of loneliness with commendable accuracy. This predictive capability shows great potential for early identification and intervention in school and clinical settings, potentially leading to timely support for those in need. Notably, our findings highlight peer communication, internet addiction, and subjective well-being as primary risk factors, offering valuable insights for policymakers looking to tackle youth loneliness. We recommend that future models be adapted based on our results to improve clinical practice and better target interventions, ultimately fostering healthier environments for children and adolescents.

## Figures and Tables

**Table 1:** Basic characteristics.

Characteristic	Total, *n* (%) (*N* = 822)	Loneliness, *n* (%) (*N* = 109)	No loneliness, *n* (%) (*N* = 713)
Age (mean (SD))	13.52 (1.14)	13.78 (1.23)	13.48 (1.13)
Gender			
Male	385 (46.84)	57 (52.29)	328 (46.00)
Female	437 (53.16)	52 (47.71)	385 (54.00)
Grade			
7	234 (28.47)	30 (27.52)	204 (28.61)
8	244 (29.68)	25 (22.94)	219 (30.72)
9	220 (26.76)	23 (21.10)	197 (27.63)
10	124 (15.09)	31 (28.44)	93 (13.04)
Relationship between parents			
Excellent	457 (55.60)	38 (34.86)	419 (58.77)
Good	223 (27.13)	29 (26.61)	194 (27.21)
Average	115 (13.99)	32 (29.36)	83 (11.64)
Not very good	21 (2.55)	8 (7.34)	13 (1.82)
Poor	6 (0.73)	2 (1.83)	4 (0.56)
Only child (*n* = 821)			
Yes	284 (34.59)	45 (41.28)	239 (33.57)
No	537 (65.41)	64.(58.72)	473. (66.43)
Weekday screen time (minutes, Mean (SD), *n* = 821)	100.38 (257.13)	127.95 (224.65)	96.16 (261.63)
Weekend screen time (minutes, Mean (SD), *n* = 820)	180.29 (195.88)	223.96 (233.42)	173.67 (188.85)
Father death (*n* = 821)			
Yes	10 (1.22)	2 (1.85)	8 (1.12)
No	811 (98.78)	106 (98.15)	705 (98.88)
Mother death (*n* = 821)			
Yes	3 (0.37)	0 (0.00)	3 (0.42)
No	818 (99.63)	108 (100)	710 (99.58)
Parents’ divorce (*n* = 821)			
Yes	74 (9.01)	13 (12.04)	61 (8.56)
No	747 (90.99)	95 (87.96)	652 (91.44)
Father works in another city (*n* = 820)			
Yes	67 (8.17)	9 (8.33)	58 (8.15)
No	753 (91.83)	99 (91.67)	654 (91.85)
Mother works in another city (*n* = 820)			
Yes	20 (2.44)	4 (3.70)	16 (2.25)
No	800 (97.56)	104 (96.30)	696 (97.75)
Number of persons living together			
0	1 (0.12)	0 (0.00)	1 (0.14)
1	47 (5.72)	9 (8.26)	38 (5.33)
2	291 (35.40)	41 (37.61)	250 (35.06)
3	292 (35.52)	38 (34.86)	254 (35.62)
4	133 (16.18)	11 (10.09)	122 (17.11)
5	50 (6.08)	9 (8.26)	41 (5.75)
6	7 (0.85)	1 (0.92)	6 (0.84)
7	1 (0.12)	0 (0.00)	1 (0.14)
Secondhand smoke			
Yes	433 (52.68)	59 (54.13)	374 (52.45)
No	389 (73.32)	50 (45.87)	339 (47.55)
Myopia (*n* = 821)			
No myopia	339 (41.29)	50 (45.87)	289 (40.59)
Myopia in the left eye	29 (3.53)	3 (2.75)	26 (3.65)
Myopia in the right eye	43 (5.24)	6 (5.50)	37 (5.20)
Myopia in both eyes	410 (49.94)	50 (45.87)	360 (50.56)
Height (mean (SD))	160.16 (7.86)	159.89 (7.09)	160.19 (7.96)
Weight (mean (SD))	53.59 (12.47)	53.79 (13.38)	53.56 (12.36)
Waist circumference (mean (SD))	72.59 (9.85)	72.05 (10.34)	72.66 (9.79)
Hip circumference (mean (SD))	87.62 (8.82)	87.20 (9.52)	87.67 (8.73)
Sexual characteristics			
Yes	524 (63.75)	55 (50.46)	469 (65.78)
No	298 (36.25)	54 (49.54)	244 (34.22)
Peer trust (mean (SD), *n* = 816)	36.84 (7.09)	30.97 (7.52)	37.73 (6.58)
Peer communication (mean (SD), *n* = 820)	27.55 (6.24)	22.35 (5.88)	28.35 (5.91)
Peer alienation (mean (SD), *n* = 810)	24.55 (4.37)	21.48 (4.28)	25.03 (4.19)
Internet addiction (mean (SD), *n* = 804)	42.73 (13.23)	51.48 (14.38)	41.37 (12.51)
Index of general affect (mean (SD), *n* = 820)	42.01 (12.05)	31.99 (12.70)	43.55 (11.18)
Index of life satisfaction (mean (SD), *n* = 821)	5.44 (1.51)	4.46 (1.75)	5.58 (1.42)

Note: This table reflected the basic characteristics prior to the imputation of missing values.

**Table 2:** Classifier information.

Classifier	Description
Random forest [43]	Random forest generates a diversified set of decision trees by randomly picking features and bootstrap aggregating (bagging). The ultimate forecast is generated by averaging or voting on each tree projections.
XGBoost [44]	XGBoost is a gradient boosting method that creates a powerful prediction model by combining weak learners (decision trees). It optimizes the objective function by adding new weak learners repeatedly that focus on the residual mistakes of prior models.
Logistic regression [45]	Logistic regression evaluates the likelihood of an event occurring depending on input factors. Maximum likelihood estimation is used by the model to learn the appropriate weights for the input characteristics.
Neural network [46]	Neural networks are a collection of artificial neurons that are interconnected to replicate the structure and function of the human brain. They are made up of three layers, input, hidden, and output, with each neuron executing a weighted sum of inputs followed by an activation function.
Support vector machine [47]	Support vector machine creates a hyperplane or a series of hyperplanes to optimize the margin between various classes, aiming for the greatest separation possible.

XGBoost = extreme gradient boosting.

**Table 3:** Classifier characteristics.

Classifier	Caret Label	R Package	Tuned Hyperparameters
Random forest	rf	randomForest	mtry
XGBoost	xgbTree	xgboost	nrounds, max_depth, eta, gamma, colsample_bytree, min_child_weight, subsample
Logistic regression	glm	glmnet	
Neural network	nnet	nnet	size, decay
Support vector machine	svmRadial	Kernlab	σ, C

XGBoost = extreme gradient boosting.

**Table 4:** Model performance on test set.

Classifier	AUC (95%CI)	*p* Value ^a^	Accuracy (95%CI)	Sensitivity	Specificity	PPV	NPV	F1
Random forest	0.85 (0.77, 0.92)	0.14	0.84 (0.79, 0.89)	0.73	0.86	0.42	0.96	0.53
XGBoost	0.87 (0.80, 0.93)	0.05	0.84 (0.79, 0.89)	0.70	0.86	0.41	0.95	0.52
Logistic regression	0.80 (0.70, 0.89)	Ref	0.74 (0.69, 0.80)	0.67	0.76	0.27	0.94	0.39
Neural network	0.80 (0.71, 0.89)	0.93	0.78 (0.72, 0.83)	0.67	0.80	0.31	0.95	0.43
Support vector machine	0.79 (0.79, 0.89)	0.83	0.76 (0.70, 0.81)	0.67	0.77	0.29	0.94	0.40

AUC = area under the receiver operating curve; PPV = positive predictive value; NPV = negative predictive curve; PLR = positive likelihood ratio; NLR = negative likelihood ratio; DOR = diagnostic odds; Ref = reference. ^a^ *p* value is the results of DeLong test of AUC curve of different machine learning models comparing with logistic regression model.

## Data Availability

Data available on request due to restrictions.

## Supplementary Materials

The following supporting information can be downloaded at: https://www.mdpi.com/article/10.3390/bs14100947/s1, Table S1: The variables and their values used in the machine learning model.

## Abbreviations

ML	machine learning
XGBoost	machine learning algorithm extreme gradient boosting
RF	random forest
CV	cross-validation
AUC	area under the receiver operating characteristic curve
PPV	positive predictive value
NPV	negative predictive value
