# Supplementary material for: Predicting the Risk of Loneliness in Children and Adolescents: A Machine Learning Study
Source: Behav Sci (Basel). 2024 Oct 15;14(10):947. doi: 10.3390/bs14100947 (PMC11504542; doi:10.3390/bs14100947)
Supplement: Supplementary file 1 [file behavsci-14-00947-s001.zip › behavsci-3222934-supplementary.pdf]

Table S1: The variables and their values used in the machine learning models

Variables	Value[1]
Lonely	1 = "Yes" (109 (13.26%)), 2 = "No"
Gender	1 = "Male" (385 (46.84%)), 2 = "Female"
Grade	Range :7-10
Class	Range :1-64
Self-report relationship with parents	1 = "Excellent"(457 (55.60%)), 2 = "Good"(223 (27.13%)),<br>3 = "Average" (115 (13.99%)), 4 = "Not very good"(21 (2.55%)) and 5 = "Poor"(6 (0.73%))
Knowledge of puberty	1 = "Know quite a lot"(150 (18.25%)), 2 = "Know a little"(639 (77.74%)), 3 = "Do not know"(33 (4.01%))
Self-reported height growth	1 = "Grows about the same each year" (461 (56.08%)),<br>2 = "Just starting to accelerate" (52 (6.33%)),<br>3 = "Has been accelerating for some time" (147(17.88%)),<br>4 = "Seems to have stopped" (162 (19.71%)),
Self-reported level of development	1 = "Develops much earlier than peers" (15 (1.82%)),<br>2 = "Develops slightly earlier than peers" (151(18.37%)),<br>3 = "Develops at the same rate as peers" (578(70.32%)),<br>4 = "Develops later than peers" (78 (9.49%))
Only child	1 = "No"(538 (65.45%)), 2 = "Yes"
Weekday Screen Time	Average minutes/day, 100.33 (256.98)
Weekend screen time	Average minutes/day, 180.29 (195.64)
Parents' divorce	1 = "Yes"(74 (9.00%)), 2 = "No"
Father works in another city	1 = "Yes"(67 (8.15%)), 2 = "No"
Number of persons living together	Range: 0-7
Secondhand smoke	0= "No" (389 (73.32%)), 1= "No"
Whether to use camphor pills	1 = "Yes"(66 (8.03%)), 2 = "No"
Myopia	1 = "No myopia" (339 (41.24%)),<br>2 = "Myopia in the left eye" (29 (3.53%)),<br>3 = "Myopia in the right eye" (44 (5.35%)),<br>4 = "Myopia in both eyes" (410 (49.88%))
Air freshener	1 = "Yes" (120 (14.60%)), 2 = "No"
Insecticide	1 = "Yes" (188 (22.87%)), 2 = "No"
Whether to use hair straightening cream	1 = "Yes" (86 (10.46%)), 2 = "No"
Use of cosmetics	1 = "Yes" (189 (22.99%)), 2 = "No"

Variables	Value[1]
Height	Physical measurement (cm), 160.16 (7.86)
Waist circumference	Physical measurement (cm), 72.59 (9.85)
Sexual characteristics	1 = "Yes" (524 (63.75%)), 0= "No"
Peer Communication	The scores for peer communication (8 items), 27.55 (6.24)
Peer Alienation	The scores for peer alienation (7 items), 24.56 (4.34)
Internet addiction	The total score of the Internet addiction scale, 42.70 (13.10)
Index of general affect	The total score of eight emotional items of the well-being scale, 42.06 (11.95)
Index of life satisfaction	The total score of two items of the well-being scale, 5.44 (1.50)

Note: [1] n(%) or Mean (SD) was used to describe variables. This table reflected the variables used in machine learning after the imputation of missing values.
